# Emerging Roles of the TRIM E3 Ubiquitin Ligases MID1 and MID2 in Cytokinesis

**DOI:** 10.3389/fphys.2019.00274

**Published:** 2019-03-19

**Authors:** Melania Eva Zanchetta, Germana Meroni

**Affiliations:** Department of Life Sciences, University of Trieste, Trieste, Italy

**Keywords:** ubiquitination, MID1, MID2, TRIM E3 ligase, cytokinesis, X-linked Opitz syndrome

## Abstract

Ubiquitination is a post-translational modification that consists of ubiquitin attachment to target proteins through sequential steps catalysed by activating (E1), conjugating (E2), and ligase (E3) enzymes. Protein ubiquitination is crucial for the regulation of many cellular processes not only by promoting proteasomal degradation of substrates but also re-localisation of cellular factors and modulation of protein activity. Great importance in orchestrating ubiquitination relies on E3 ligases as these proteins recognise the substrate that needs to be modified at the right time and place. Here we focus on two members of the TRIpartite Motif (TRIM) family of RING E3 ligases, MID1, and MID2. We discuss the recent findings on these developmental disease-related proteins analysing the link between their activity on essential factors and the regulation of cytokinesis highlighting the possible consequence of alteration of this process in pathological conditions.

## Introduction

### Cytokinesis and Ubiquitination

Cytokinesis is the final step of cell division that consists in the physical separation into two cells after nuclear and cytoplasmic content portioning. It requires coordinated actions of the cytoskeleton, membrane systems, and cell cycle engine, which are precisely controlled in space and time. Cytokinesis starts after anaphase and consists of different steps: central spindle assembly, division plane specification, contractile ring assembly, cytokinetic furrow ingression, midbody appearance, and abscission (Echard et al., 2004; Eggert et al., 2006). Following abscission, the residual midbody is either released in the extracellular medium, degraded by selective autophagy or persists in the cytoplasm of one daughter cell (Agromayor and Martin-Serrano, 2013). Interestingly, inherited midbodies can affect cell polarity, cell communication, stemness (Bernabe-Rubio et al., 2016; Antanaviciute et al., 2018; Li et al., 2018). Cytokinesis failure leads to defective mitosis and high chromosomal instability. Thus, for proper organisms growth and development a correct cell division is essential (D’Avino et al., 2015).

The activity of cytokinesis factors must be precisely orchestrated and oscillates by regulated post-translational modifications such as ubiquitination. Covalent conjugation of ubiquitin to a substrate is operated through sequential action of activating (E1), conjugating (E2), and ligase (E3) enzymes. Importantly, the E3 ubiquitin ligases recognise the specific substrates to be ubiquitinated (Komander and Rape, 2012). Ubiquitination is long known for driving cell cycle transitions. For example, metaphase-to-anaphase transition is triggered by the E3 ligase APC/C^CDC20^ that promotes the degradation of cyclin B and securin, allowing mitotic exit (Teixeira and Reed, 2013). Ubiquitination is a signal not only for protein degradation but also for non-proteolytic fate through the building of chains with different ubiquitin linkages and topologies (Kulathu and Komander, 2012). As example, the E3 CRL3^KLH21^ mono-ubiquitinates Aurora B allowing its MKLP2-mediated translocation to promote correct kinetochore–microtubule attachments during metaphase (Krupina et al., 2016). Further, a giant protein possessing E2/E3 activity, BRUCE, interacts with midbody components affecting the distribution of ubiquitin at the midbody site, in that being fundamental for abscission (Pohl and Jentsch, 2009).

Many other E3 ligases have been described to dynamically control cell cycle events through both proteolytic and non-proteolytic signals (Gilberto and Peter, 2017). The TRIpartite Motif (TRIM) family is the major sub-class of RING-E3 ubiquitin ligases counting over 70 members implicated in several physiological and pathological processes (Reymond et al., 2001; Meroni and Diez-Roux, 2005; Short and Cox, 2006; Hatakeyama, 2017). Here, we will focus on the role of two members of this family, TRIM18/Midline1/MID1 and TRIM1/Midline2/MID2 (from here onward MID1 and MID2), in cytokinesis.

### MID1 and MID2 E3 Ubiquitin Ligases

Among the TRIM family, MID1, and MID2 are very close paralogues originating from a common ancestor after the invertebrate/vertebrate separation and predating the bony vertebrates appearance (Sardiello et al., 2008). Consistently, human *MID1* and *MID2* genes have a conserved genomic structure, are both located on the X chromosome, and share a high degree of identity (70%) at nucleotide level (Quaderi et al., 1997; Buchner et al., 1999). This similarity is patent also in their domain structure. MID1 and MID2 present the N-terminal hallmark of the TRIM family, the tripartite motif, composed of the catalytic RING domain followed by tandem B-Box 1 and B-box 2 and a Coiled-coil region. The TRIM family is further subdivided into 9 classes (C-I to C-IX) according to the domains present C-terminal to the tripartite module with the SPRY-containing C-IV subfamily being the most numerous (Reymond et al., 2001; Short and Cox, 2006). MID1 and MID2 C-terminus displays a COS domain, a Fibronectin type III repeat and a PRY-SPRY domain as all C-I sub-family TRIM members (Reymond et al., 2001; Short and Cox, 2006; Table 1). While the Fibronectin type III repeat and PRY-SPRY domain role in MID proteins is unclear, the COS domain was shown to mediate MID1 and MID2 association with the microtubular apparatus (Buchner et al., 1999; Cainarca et al., 1999; Short and Cox, 2006). Microtubular binding of MID1 is detectable also during mitosis and on the central spindle and midbody during cytokinesis (Cainarca et al., 1999). Recently, localisation at the midbody was reported also for MID2 (Gholkar et al., 2016). Whether MID proteins co-localise at the midbody in a mutual manner is still not unravelled. The coiled-coil region of MID1, besides mediating self-interaction, is also responsible for hetero-interaction with MID2 (Short et al., 2002; Meroni and Diez-Roux, 2005). The extent and stoichiometry of MID1/MID2 interaction is at present not known but one can envisage functions elicited by either homo- or hetero-complexes resulting in partial functional redundancy between MID proteins. Indeed, redundancy between the chicken orthologues of *MID* genes, *cMid1*, and *cMid2*, has been reported during the determination of avian left/right axis (Granata et al., 2005). Intriguingly, both human genes are implicated in genetic diseases: *MID1* is mutated in patients presenting a complex neurodevelopmental disorder, the X-linked Opitz G/BBB syndrome (OS) (OMIM 300000) (Quaderi et al., 1997); and *MID2* is mutated in an X-linked intellectual disability (OMIM 300928) (Geetha et al., 2014). This further suggests MID1 and MID2 overlapping functions. Along the same line, analyses of these genes during embryonic development in human, mouse and chicken show partial overlapping expression. *MID1* is mainly found in the central nervous system (CNS), the developing branchial arches, the gastrointestinal and the urogenital systems, and the developing heart correlating with the tissues affected in OS (Dal Zotto et al., 1998; Richman et al., 2002; Pinson et al., 2004). *MID2* displays low embryonic expression in the developing CNS, thymus and heart (Buchner et al., 1999). On the contrary, in human adult tissues, *MID1* and *MID2* have a distinct expression pattern: *MID2* is mainly expressed in thyroid, smooth muscle, prostate, breast, and ovary whereas *MID1* is found in the cerebellum, lung, colon, urinary bladder, prostate, placenta, breast, and ovary and retina (source ^1^^,^^2^ ).

**Table 1:** Summary of principal MID1 and MID2 features.

Official symbol	MID1	MID2
Official name	midline 1	midline 2
Gene ID	4281	11043
Aliases	FXY, MIDIN, TRIM18, RNF59	FXY2, TRIM1
Location	Xp22.2	Xq22
CDS length	2,004 nt	2,148 nt
Protein length	667 aa	715 aa
Associated Syndromes	X-linked Opitz G/BBB syndrome (OMIM #300000)	Mental retardation, X-linked (OMIM #300928)
Protein domains	RING domain; B-box type 1 and 2; coiled-coil; COS domain; fibronectin type 3 domain; PRY/SPRY domain	RING domain; B-box type 1 and 2; coiled-coil; COS domain; fibronectin type 3 domain; PRY/SPRY domain
Protein function	E3 ubiquitin ligase	E3 ubiquitin ligase
Subcellular location/component (UniProt)	cytosol, microtubule, spindle (www.uniprot.org/uniprot/O15344)	cytosol, microtubule, exosome (www.uniprot.org/uniprot/Q9UJV3)
Amino acid modification (UniProt)	Phosphoserine 92, 96, 511 (www.uniprot.org/uniprot/O15344)	Phosporylated on serine and threonine residues (www.uniprot.org/uniprot/Q9UJV3)
Interactors (common interactors are indicated in bold and the relative references are listed)	**MID1**, **MID2** (Short et al., 2002); **ALPHA-4**, PPP2CB, PPP2CA, PPP2R1A (Liu et al., 2001; Watkins et al., 2012); PTPA (Du et al., 2014); **ASTRIN** (Gholkar et al., 2016); BRAF35 (Zanchetta et al., 2017); MID1IP1 (Berti et al., 2004); ANXA2, EEF1A1, NPM1, HSP90AA1, RACK1, RPS3, RPS8; (Aranda-Orgilles et al., 2008b); PAX6 (Pfirrmann et al., 2016); STK36 (Schweiger et al., 2014); TRIM16 (Bell et al., 2012); **TUBB, TUBB4B** (Gholkar et al., 2016); UBC (Trockenbacher et al., 2001); **UBE2D1**, **UBE2D2**, **UBE2D3, UBE2D4**, **UBE2E1**, **UBE2E2, UBE2E3**, **UBE2N** (Napolitano et al., 2011)	**MID2**, **MID1** (Short et al., 2002); **ALPHA-4** (Short et al., 2002); **ASTRIN**, ASPM, CEP128 (Gholkar et al., 2016); LNX1 (Lenihan et al., 2017); TRIM27, TRIM42, TRIM54 (Rolland et al., 2014); TRIM29, TRIM32 (Reymond et al., 2001); **TUBB**, **TUBB4B** (Gholkar et al., 2016); **UBE2D1**, **UBE2D2**, **UBE2D3**, **UBE2D4**; **UBE2E1**; **UBE2E2**, **UBE2E3**, **UBE2N**; (Napolitano et al., 2011)


Regarding their E3 ligase function, *in vitro* activity for both MID1 and MID2 has been described (Han et al., 2011; Napolitano et al., 2011). In more physiological contexts, both unique and common MID proteins partners have been identified, some of which are reported as MID E3 ligases *bona fide* substrates. These data are briefly summarised in Table 1 and recently thoroughly reviewed in Li et al. (2016); Winter et al. (2016). These findings suggest that the two TRIM paralogues evolved maintaining common roles while developing their own specificity, likely in a context-specific manner. Their expression analyses during embryonic development revealed a preference for mitotically active compartments suggesting a role during cell cycle progression and here below we will discuss recent findings that support a role of MID1 and MID2 during the cytokinetic process.

## MID1 and MID2 Involvement in Cytokinesis

As mentioned above, recent reports suggest an involvement of MID1 and MID2 in cytokinesis. Indeed, in HeLa cells, the depletion of either MID1 or MID2 leads to cell division defects, namely, cytokinetic arrest often followed by cell death and delay or failure to divide with regression into binucleated cells (Gholkar et al., 2016). This role is likely elicited through the interaction with several partners that we discuss here below.

### Astrin

A recent work uncovered that both MID1 and MID2 bind the microtubule-associated protein Astrin (also known as SPAG5) (Gholkar et al., 2016). Astrin is important in the regulation of mitotic progression since its depletion causes centrosome instability and mitotic spindle malformation in HeLa cells. Astrin associates with the spindle throughout mitosis allowing chromosome alignment and segregation (Mack and Compton, 2001; Gruber et al., 2002). Diverse kinases, such as GSK3, Aurora A and Plk1, phosphorylate Astrin to regulate its mitotic function during spindle assembly (Cheng et al., 2008; Chiu et al., 2014; Chung et al., 2016).

The interaction between Astrin and the two TRIM proteins occurs independently from the cell cycle but has consequences only on cytokinesis. MID1 and MID2 partially co-localise with Astrin at the midbody of dividing cells. Interestingly, MID2 alone promotes Astrin ubiquitination at a unique site (K409) at mitotic exit targeting the protein to proteasomal degradation. Inappropriate accumulation of Astrin at the midbody provokes cytokinetic arrest, increased binucleation and cell death. Consistently, MID2 depletion leads to minor defects in early mitosis and major defects in cytokinesis supporting the importance of its E3 ligase activity in regulating Astrin degradation (Gholkar et al., 2016).

Unexpectedly, also MID1-deprived cells display division defects, including cytokinetic arrest, delayed or aborted abscission, inducing cell binucleation or death (Gholkar et al., 2016). At present it is not known if the observed cytokinetic phenotype is related to the lack of MID1-Astrin association and which is the mechanism involved. Further, whether MID1, MID2, and Astrin form a single protein complex is still undefined. An intriguing possibility is that distinct and dynamic homo- or hetero-MID complexes exist to target not only Astrin but also other cytokinesis-related proteins.

### Alpha4/PP2Ac

The first reported target of MID1 E3 ligase activity was the catalytic subunit of serine/threonine protein phosphatase 2A (PP2Ac) driven to ubiquitin-mediated proteasomal degradation (Trockenbacher et al., 2001). MID1 directly interacts through the B-box 1 domain with Alpha4 (α4) that is one of the atypical regulatory subunits of PP2A (Nanahoshi et al., 1998; Liu et al., 2001; Trockenbacher et al., 2001; Short et al., 2002; LeNoue-Newton et al., 2011). Later on, α4 was reported to be a MID1 substrate as well (Watkins et al., 2012; Du et al., 2013). Active PP2A is a heterotrimeric holoenzyme composed of a catalytic (C subunit), a scaffold (A subunit) and a variable regulatory subunit (B, B′, B′′, or B′′′ subunits) that dictate substrate selectivity and subcellular localisation of the phosphatase holoenzyme. A small pool of PP2Ac was shown to form a complex containing α4 instead of the B subunit (Baskaran and Velmurugan, 2018). At cytokinesis, PP2Ac, A and B′γ1 subunits are all localised at the midbody in HeLa cells (Wu et al., 2017). In addition, PP2A-B′ holoenzyme counteracts Aurora B kinase activity controlling the length of spindle midzone through KIF4A dephosphorylation (Bastos et al., 2014).

The mechanism of self-regulation of the MID1/α4/PP2Ac complex involves a series of ubiquitination and dephosphorylation events that have been long studied but still remain to be completely unravelled. Initially, α4 was described to protect PP2Ac from degradation. Although *in vitro* assays suggested that MID1 catalyses mono- and di-ubiquitination of PP2Ac it is likely that other E3 ligases synergistically or alternatively are required to target its proteasomal degradation (Watkins et al., 2012; Du et al., 2014). Interestingly, MID1 not only targets a sub-pool of α4 for poly-ubiquitination-mediated degradation but also mono-ubiquitinates α4, triggering calpain mediated cleavage and degradation of its C-terminus containing the MID1 binding region (Watkins et al., 2012; Du et al., 2013). Whatever the mechanism, α4 cleavage disrupts the MID1/α4/PP2Ac complex, influencing PP2Ac stability (Winter et al., 2016). Altered PP2Ac activity affects mTORC1 complex formation and signalling (Liu et al., 2011). This pathway can play a significant role in the pathogenesis of OS and it would be interesting to investigate a possible MID1-mediated mTORC1 involvement in cytokinesis.

MID proteins contain two conserved phosphorylation consensus sites (Ser92 and Ser96) for GSK3 and MAPK, respectively (Short et al., 2002). Interestingly, MID1 interaction with α4 results in PP2Ac recruitment on microtubules and MID1 dephosphorylation at Ser96 (Aranda-Orgilles et al., 2008a). It is tempting to speculate that a similar regulatory mechanism involves MID2 as it binds α4 as well (Short et al., 2002). A fine balance of MID1 phosphorylation and dephosphorylation via MAPK and PP2A is important for regulating its affinity and its bi-directional movement along the microtubule network through kinesins and dyneins (Liu et al., 2001; Trockenbacher et al., 2001; Aranda-Orgilles et al., 2008a). Whether MID1 phosphorylation status affects E3 ligase activity or influences the interaction with α4 have not been addressed yet.

These findings leave some questions open and some issues still controversial. Indeed, α4 was shown to serve as a binding partner of PP2Ac rendering the latter catalytically inactive to avoid improper protein dephosphorylation. Then, when needed, α4 contributes to stabilise newly synthetised PP2Ac preventing its ubiquitin-mediated degradation thus permitting its assembly into functional PP2A holoenzymes (Kong et al., 2009). It is possible that binding of α4 to MID1 is needed to preserve a pool of newly available PP2Ac that can be transported along the microtubules to the spindle midzone. At this point, PP2Ac might become available for incorporation into active PP2A to dephosphorylate a pool of microtubule-associated phosphoproteins, such as KIF4A, to precisely control cytokinesis.

### BRAF35

A recently identified MID1 substrate is the BRCA2-associated factor BRAF35 (also known as HMG20B) (Zanchetta et al., 2017) that was first isolated as part of a large nuclear multi-protein complex containing BRCA2 (Marmorstein et al., 2001).

MID1 ubiquitinates BRAF35 and is necessary for its turnover mainly outside the nucleus. Strikingly, although BRAF35 protein levels are regulated by the proteasome, atypical linkages are preferred in MID1-mediated ubiquitination, involving K6, K27, and K29 poly-ubiquitin chains. Among them, only K6 poly-ubiquitination promotes BRAF35 proteasomal degradation while K27 and K29 chains have no degradative effects (Zanchetta et al., 2017). The present knowledge does not offer insights to infer the effect of these modifications on BRAF35 (Kulathu and Komander, 2012). Of note, MID1 and BRAF35 co-localise in the cytoplasmic compartment and BRAF35 accumulates in larger cytoplasmic bodies when MID1 is depleted from HeLa cells (Zanchetta et al., 2017).

Recently BRAF35 was found in a region of the midbody compatible with MID1 localisation (Cainarca et al., 1999; Lee and Venkitaraman, 2014; Gholkar et al., 2016). Consistently, BRAF35 also associates with the previously mentioned PP2A target KIF4A, a motor protein that is essential for central spindle midzone and midbody organisation (Lee and Kim, 2003). Direct interaction between the cargo domain of KIF4A and BRCA2 was also proved, suggesting the existence of a multi-protein complex in which also BRAF35 takes part (Wu et al., 2008). BRCA2 is recruited on the midbody by the actin-binding protein Filamin A and is necessary for correct localisation of other midbody proteins, such as MKLP1, MKLP2, and PRC1 (Mondal et al., 2012). On the midbody BRCA2 forms a complex with CEP55, Alix, and Tsg101 and is required for the recruitment of the ESCRT machinery, necessary for abscission (Mondal et al., 2012). Depletion of BRAF35 from HeLa cells results in a delayed transition from anaphase to the completion of cell division (Lee et al., 2011). About half of BRAF35-depleted cells start cleavage furrowing but fail to divide, becoming binucleated (Lee et al., 2011). Interestingly, the same phenotype had previously been observed in BRCA2-deficient cells (Daniels et al., 2004). It is interesting that MID1 depletion phenocopies the cytokinesis failure-derived defects that were observed in the absence of BRAF35 or BRCA2 (Gholkar et al., 2016).

The C-terminal portion of BRAF35 spanning aa 173–276 is the minimal fragment required for BRCA2 binding and is also sufficient for its midbody localisation (Lee and Venkitaraman, 2014). However, contrary to the entire C-terminal fragment (aa 173–317), expression of the 173–276 aa fragment fails to restore cytokinesis in BRAF35-depleted cells suggesting the need of an additional factor (Lee and Venkitaraman, 2014). MID1 could represent such interactor, as the binding to BRAF35 occurs in an overlapping region (aa 230–317) to that necessary to form the BRAF35/BRCA2 complex, thus contributing to proper cytokinesis (Zanchetta et al., 2017).

**FIGURE 1:**
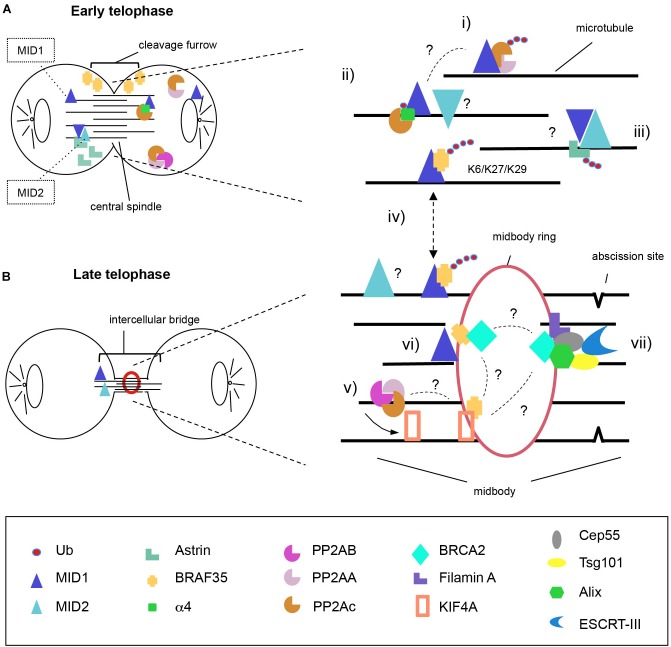
Model for MID1 and MID2 complexes distribution during cytokinesis. MID1 and MID2 localise on the microtubules both at early **(A)** and late **(B)** telophase where they possibly hetero-interact. We propose action of MID proteins during both steps. At the central spindle, MID1 poly-ubiquitinates PP2Ac to regulate PP2A levels (i) and mono-ubiquitinates a4 to disrupt the association of a4-PP2Ac (ii). Available PP2Ac can be assembled into active PP2A holoenzymes that dephosphorylate KIF4A to control the length of spindle midzone (v). At early telophase, MID2 ubiquitinates Astrin inducing its proteasomal degradation and removal from the intercellular bridge in order to allow completion of cytokinesis (iii). BRAF35 abundance and localisation at the intercellular bridge is regulated through MID1-dependent ubiquitination using non-canonical ubiquitin linkages (K6, K27, and K29) (iv); there, BRAF35 associates with KIF4A and/or BRCA2 (vi); (vii) BRCA2 is recruited to the midbody through Filamin A and forms a complex with Cep55, Alix and Tsg101, allowing the recruitment of ESCRT-III to complete abscission. It is still unknown to what extent MID proteins activity on these substrates is interconnected and this is highlighted in the model with question marks (?). One intriguingly possibility is that KIF4A might represent the central player linking all the complexes regulate by MID1 and MID2.

The findings discussed here support the role of MID proteins in cell division through activities on multiple targets likely not only promoting their proteasomal degradation. However, it is still not clear to what extent their E3 ligase activity on the substrates cited in this review and graphically summarised in Figure 1 are interconnected. In this model, KIF4A might play a central role in the MID1- and MID2-regulated network. It is a matter of fact that both MID1 and MID2 are needed for successful cytokinesis with consequences in physiological and pathological conditions.

## Conclusion

Although tightly regulated, cytokinesis lacks an effective checkpoint to ensure its fidelity. Cytokinesis can fail at different steps, because cleavage furrow ingression is inhibited or incomplete, or abscission is defective. The originated cells show increased chromosomal instability resulting in the generation of multipolar spindles and chromosome segregation defects (Sagona and Stenmark, 2010). Errors in cytokinesis may thus have dramatic consequences ranging from embryonic defects to cancer. Aberrant expression of cytokinesis regulators is indeed largely associated with many cancer types (Lens and Medema, 2019).

Recent findings showed elevated MID1 expression in prostate cancer and alteration of the MID1/a4/PP2A axis in lung adenocarcinoma and MID1 expression levels positively correlate with tumour Gleason scores (Kohler et al., 2014; Zhang et al., 2018). Similarly, high level of MID2 expression was significantly correlated with breast cancer progression (Wang et al., 2016). On the contrary, down-regulation of MID1 mediated by miR-135b has been shown to promote tumour progression of mammary carcinomas (Arigoni et al., 2013). Of note, high levels of Astrin have been described in cervical, pancreatic, hepatocellular carcinoma, and non-small-cell lung cancers (Valk et al., 2010; Yuan et al., 2014; Ansari et al., 2015; Liu et al., 2018; Yang et al., 2018). In the case of BRAF35, the A247P mutation reported in a case of lung carcinoma was shown to interfere with its midbody localisation and BRCA2 binding (Lee and Venkitaraman, 2014). This mutation induces cytokinesis failure through a dominant negative mechanism possibly affecting MID1 activity. Thus, it appears that dysregulation of MID1 plays a role in tumourigenesis, likely affecting factors that control somatic cell proliferation.

Clinically, MID1 and MID2 are implicated also in genetic developmental disorders (Quaderi et al., 1997; Geetha et al., 2014). Their involvement in cytokinesis does not come as a surprise as embryonic development is the organism phase with the highest mitotic index. During development, aberrant cytokinesis can have a strong impact not only on cell proliferation but also on morphogenetic processes. In fact, inherited midbodies can affect cell polarity and cell communication and, in epithelia, midbody positioning influences planar tissue architecture (Herszterg et al., 2014; Bernabe-Rubio et al., 2016; Antanaviciute et al., 2018).

The identification of the involvement of MID1 and MID2 in cytokinesis is intriguingly though we are still far from clarifying the precise dynamics of the occurring events. Further investigations will be necessary to understand the dynamics of the complexes containing MID proteins and Astrin, BRAF35 and PP2A and their interplay, if any. The future dissection of these mechanisms, together with parallel *in vivo* studies, will be necessary to get a comprehensive picture and for future clinical application.

## Author Contributions

MZ and GM conceived the study, drafted the manuscript, and reviewed and edited the manuscript. GM acquired the funding.

## Conflict of Interest Statement

The authors declare that the research was conducted in the absence of any commercial or financial relationships that could be construed as a potential conflict of interest.

## Footnotes

1 www.proteinatlas.org

2 www.ncbi.nlm.nih.gov/gene
